# Modelling the surface free energy parameters of polyurethane coats—part 1. Solvent-based coats obtained from linear polyurethane elastomers

**DOI:** 10.1007/s00396-012-2826-4

**Published:** 2012-11-08

**Authors:** Piotr Król, Jaromir B. Lechowicz, Bożena Król

**Affiliations:** Department of Polymer Science, Faculty of Chemistry, Rzeszów University of Technology, Al. Powstańców Warszawy 6, 35-959 Rzeszów, Poland

**Keywords:** Synthesis of the polyurethane coatings, NMR spectroscopy, Contact angles, Owens–Wendt method, Prediction of the surface free energy parameter, Additive model of the surface free energy

## Abstract

Polyurethane elastomers coating were synthesised by using typical diisocyanates, polyether and polyester polyols and HO-tertiary amines or diols as a chain extenders. Mole fractions of structural fragments (*κ*_exp_) responsible for the polar interactions within polyurethane chains were calculated by ^1^H NMR method. Obtained results were confronted with the analogous parameter values (*κ*_theor_) calculated on the basis of process stoichiometry, considering the stage of the production of isocyanate prepolymers and reaction of their extension for polyurethanes. Trials of linear correlation between the *κ*_exp_ parameters and surface free energy (SFE) values of investigated coatings were presented. SFE values were determined by Owens–Wendt method, using contact angles measured with the goniometric method. Based on achieved results, another empirical models, allowing for prediction the influence of the kind of polyurethane raw materials on SFE values of received coatings were determined. It was found that it is possible to regulate the SFE in the range millijoules per cubic metre by the selection of appropriate substrates. It has been found that use of 2,2,3,3-tetrafluoro-1,4-butanediol as a fluorinated extender of prepolymer chains is essential to obtain coatings with increased hydrophobicity, applied among others as biomaterials—next to diphenylmethane diisocyanate and polyoxyethylene glycol.

Polyurethanes have for many years been used in traditional outlets like structural elastomers, foamed materials for automotive and construction industries, numerous types of protective coats, etc. Attention has been captured recently by the applicability of those materials in implantable devices in view of their excellent mechanical and biocompatible properties. However, bio-stability places limitations on the long-term use of such implants. Those applications are strictly controlled by the surface properties of the polymer material, inclusive of possible modifications of the surface free energy (SFE) value towards its reduction [1, 2]. The polyurethane material itself is a polar polymer with the SFE value exceeding 50 kJ/m^2^ in many cases which makes it biodegradable and completely immiscible in practice with polyolefins, i.e. with the materials which also take an important place in tissue engineering [3, 4]. The SFE value is of great importance, too, in the production of anti-graffiti coats and the desired reduction of SFE can be obtained by incorporation of an OH-functional silicone modified poly-acrylate additive [5]. The highest reduction in SFE of polyurethane coatings may be obtained by incorporation of apolar structures which contain fluorides, e.g. pendant fluorinated *bis* chain extender-ammonium salts, which additionally promote the antibacterial properties [6] or by incorporation of hydroxy-terminated perfluoropolyethers into polyurethane soft segments [7]. The formation of nanocomposites: polyurethane with the use of polysilsequixane (POSS) may also reduce the SFE value. The introduction of POSS into a PU system leads to high glass transition temperatures, enhanced storage moduli and improved stability, and it additionally reduces the SFE [8]. That turned out applicable in the production of polyurethane powder lacquers [9]. The above examples themselves make evidence for the high importance of SFE in the formulation of polyurethane plastics which satisfy the requirements of contemporary materials engineering. Having those questions in mind and following the progress within theoretical explanations for the effects of polyurethane structures on the SFE values of films and coatings obtained from those polyurethanes [10, 11], we made an effort within the present study to develop quantitative *structure*–*SFE* correlations for those polymers. Our research was inspired by earlier theoretical reports by van Krevelen [12] who had linked the SFE to the parachor *P*. The values of SFE (*γ*) were earlier inferred from the measured parachor values by Philips (1929) and Quayle (1953), and they put forward a relatively simple equation for that purpose [12]:

1 $$ \gamma ={{\left( {\frac{P_s}{V}} \right)}^4} $$

where:

*P*_*s*_: groups contribution of the parachor (*P*), expressed in (cm^3^/mol)x(mJ/m^2^)
***V***: liquid molar volume (cm^3^/mol)

The parachor as suggested by Sugden (1924) is counted among the so-called atomic constants, and its value for liquids is dependent on surface tension *σ*, density *d*_*c*_, density of vapour in equilibrium with that liquid *d*_*p*_ and molecular weight *M*:

2 $$ P=M\cdot \frac{{{\sigma^{0.25 }}}}{{d_c -{d_p}}} $$

As a parachor is an additive quantity, the parachor for a specific chemical structure may be calculated by summing up increments of that structure, i.e. individual atoms and functional groups, like *H*, halogen, CH_2_ group or benzene ring. The possibility of linking SFE to the parachor and the additive effects of the polar and dispersive components of SFE as provided in the Owens and Wendt theory [13], all that made us use the additive model as a tool to describe the effects of polyurethane chemical structures on the SFE values of the polymer films. Our research was preceded by the attempts to correlate SFE and the structural parameter *κ* which had been developed especially for that purpose and which described polarity of a polymer film. *κ* parameter was determined on the basis of the NMR spectra.

The first part of that paper presents possible estimation and prediction of SFE values for polyurethane films prepared with the use of organic solvents, and the research on films obtained from waterborne polyurethane cationomers will then be provided.

## Experimental

### Reagents


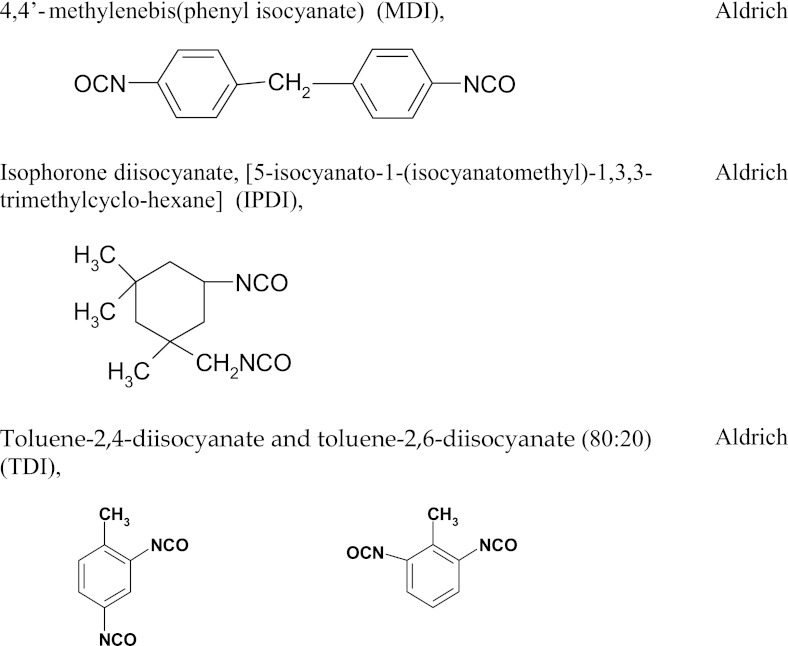

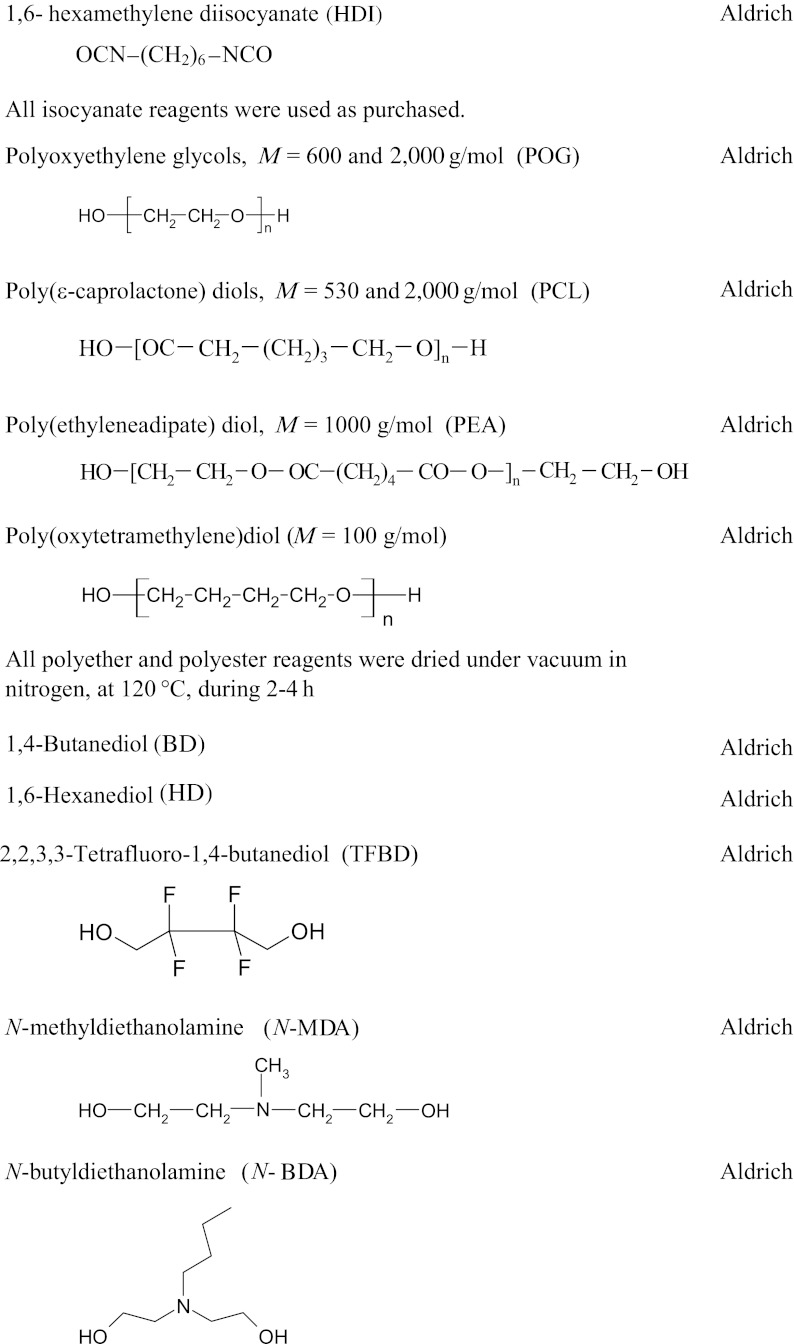

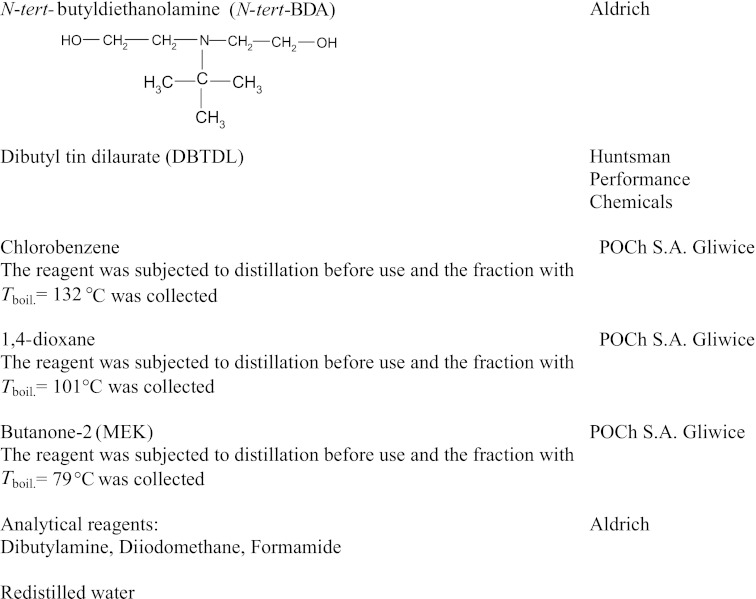


### Method for the synthesis of linear polyurethane coatings

Polyurethanes were synthesised in a two-stage polyaddition process, in a glass stand composed of: three-necked flask, heating bowl, mechanical agitator, dropping funnel, thermometer, reflux condenser and nitrogen supply nozzle. The prepolymer was synthesised at stage 1 with the use of appropriate diisocyanates and polyols at the molar ratio of 2:1. The process was conducted at 60 °C during 2–3 h, in the presence of DBTDL as a catalyst which was added at 0.1 wt.% on polyether or polyester. The reaction was terminated when the concentration of free −NCO groups as established analytically was equal to that resulting from stoichiometric calculations. That stage may be presented by the following reaction:

3 $$ A+2\;B\to BAB $$

where:

*A*: Structure derived from polyol (POG, PTMO, PEA or PCL)
*B*: Structure derived from diisocyanate (MDI, TDI, HDI or IPDI)

It should be mentioned that the chemical constitution of the so-obtained BAB prepolymers is generally dependent on the type of diisocyanate (i.e. on reactivity of its functional groups and on the substitution effects) and on the reaction conditions (i.e. temperature, type and amount of catalyst) which may for example be favourable for the formation of allophanate structures. Linear prepolymers were produced under the synthesis conditions as specified. Then they were extended at the second stage with a suitable reactant (*N*-MDA, *N*-BDA, BD, HD or TFBD, respectively) in the solution of 1,4-dioxane, chlorobenzene or MEK, at the concentration of about 40 wt.%, with the [−NCO]-to-[−OH] ratio maintained at 1:1. The chain extension reaction was conducted until all free −NCO groups disappeared completely (2–3 h). The polymer chain extension process may be illustrated by the reaction:
4where:

*Q*—structure derived from BD, HD, TFBD, *N*-MDA, *N*-BDA or *N*-*tert*-BDA

The final product obtained after prepolymer extension was the linear polyurethane as expected. Its chain was composed of structural units which formed soft polyol segments *A* and hard urethane segments. The latter were compiled of diisocyanate-derived structural fragments *B* and chain extension fragments Q which were linked together with urethane bonds NH–CO–O–(*x*):
5


PCL- or PEA-derived polyol structures in poly(ester-urethane), and PEOs in poly(ether-urethane), made soft segments in the synthesised PUs. On the other hand, hard segments were composed of the urethane segments. They were derived from diisocyanates which have not been converted at stage 1 or from prepolymer fragments with −NCO end groups and low molecular weight chain extenders.

**Scheme 1:**
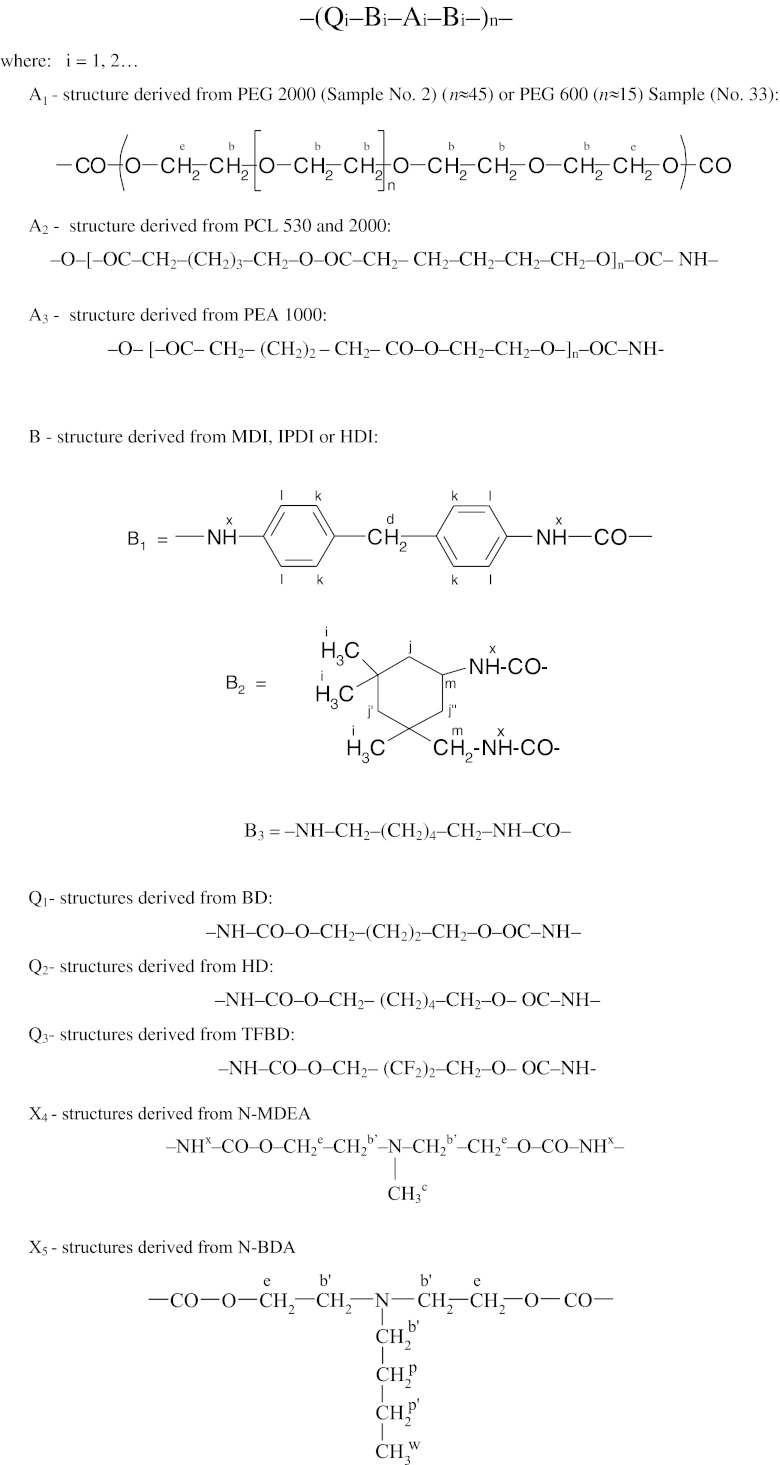
Exemplary structures of polyurethane chains

The chemical compositions of the produced polyurethanes were presented in Table 1. The exemplary chain structures in synthesised polyurethanes were presented in Scheme 1. The reference films were prepared by covering PTFE plates with the solution of a linear polyurethane (about 40 wt.%) and conservative evaporation of 1,4-dioxane, chlorobenzene or MEK (sample No. 16) in a vacuum drier, at 80 °C, over 6 h, followed by additional conditioning by exposure to ambient air during 10 days. The detailed information on polyurethane synthesis conditions was presented in our earlier paper [14]. The results of mechanical tests for these coatings confirm that the synthesised materials are elastomers and that their strength at about 40 MPa and elongation at a break of 40 % is sufficient for protective coatings. The surface shape and surface roughness are essential if the measured contact angles and their interpretation are to be correct.

**Table 1:** Chemical compositions of synthesised linear polyurethanes

Sample No.	Type of diisocyanate	Type of polyol (molecular weight)	Type of chain extender	Fluorine content (wt.%)	Type of solvent	Structural parameter	
						*κ*_theor_	*κ*_exp_
1	MDI	POG (2000)	*N*-BDA	0	Chlorobenzene	47.0	45.7
2	MDI	POG (2000)	*N*-BDA	0	1,4-dioxane	47.0	45.9
3	MDI	POG (2000)	*N*-PhDA	0	1,4-dioxane	47.4	45.1
4	MDI	POG (2000)	*N*-MDA	0	1,4-dioxane	49.3	47.3
5	MDI	POG (2000)	*N*-MDA	0	Chlorobenzene	49.5	48.0
6	MDI	POG (2000)	BD	0	1,4-dioxane	47.2	47.3
7	MDI	POG (2000)	BD	0	Chlorobenzene	46.7	35.6
8	MDI	POG (2000)	*N*-*tert*-BDA	0	1,4-dioxane	46.6	45.7
9	MDI	POG (600)	TFBD	5.70	1,4-dioxane	–	–
10	MDI	POG (600)	*N*-MDA	0	1,4-dioxane	48.3	–
11	MDI	POG (600)	HD	0	1,4-dioxane	41.0	40.0
12	MDI	PEA (1000)	*N*-MDA	0	1,4-dioxane	34.7	28.3
13	MDI	PCL (2000)	BD	0	1,4-dioxane	22.9	22.3
14	MDI	PCL (530)	TFBD	6.01	1,4-dioxane	–	–
15	MDI	PCL (530)	BD	0	1,4-dioxane	25.8	29.0
16	MDI	PTMO (1000)	BD	0	MEK	–	–
17	TDI	POG (2000)	BD	0	1,4-dioxane	48.1	49.1
18	TDI	POG (600)	TFBD	5.88	1,4-dioxane	–	–
19	TDI	POG (600)	BD	0	1,4-dioxane	46.4	41.7
20	HDI	POG (2000)	*N*-BDA	0	1,4-dioxane	49.8	47.2
21	HDI	POG (2000)	*N*-BDA	0	1,4-dioxane	49.8	46.8
22	HDI	POG (2000)	*N*-BDA	0	Chlorobenzene	49.8	46.8
23	HDI	POG (2000)	BD	0	1,4-dioxane	50.0	46.8
24	IPDI	POG (2000)	*N*-BDA	0	Chlorobenzene	46.4	41.7
25	IPDI	POG (2000)	*N*-BDA	0	1,4-dioxane	46.4	43.4
26	IPDI	POG (2000)	*N*-MDA	0	Chlorobenzene	47.3	43.5
27	IPDI	POG (2000)	HD	0	1,4-dioxane	45.7	43.3
28	IPDI	POG (2000)	HD	0	1,4-dioxane	45.7	–
29	IPDI	POG (2000)	HD	0	1,4-dioxane	46.4	42.9
30	IPDI	POG (2000)	BD	0	1,4-dioxane	46.5	43.6
31	IPDI	POG (2000)	BD	0	Chlorobenzene	46.5	42.7
32	IPDI	POG (600)	*N*-MDA	0	1,4-dioxane	46.7	35.6
33	IPDI	POG (600)	*N*-MDA	0	Chlorobenzene	46.7	43.9
34	IPDI	PEA (1000)	*N*-MDA	0	1,4-dioxane	34.5	30.0

*MDI* diphenylmethane diisocyanate, *TDI* toluene diisocyanate, *HDI* hexamethylene diisocyanate, *IPDI* isophorone diisocyanate, *POG* polyoxyethylated polyols, *PEA* poly(ethylene adipate), *PCL* polycaprolactone, *N-MDA N*-methyldiethanolamine, *N-BDA N*-butyldiethanolamine, *N-PhDA N*-phenyl-diethanolamine, *BD* 1,4-butanediol, *HD* 1,6-hexanediol, *TFBD* 2,2,3,3-tetrafluoro-1,4-butanediol, *N*-*tert*-butyldiethanolamine (*N*-*tert*-BDA), *MEK* butanone-2

### NMR spectroscopy

^1^H NMR spectra of the obtained polyurethanes were taken with the use of the FT NMR Bruker Avance 500^II^ spectrometer. The film samples (i.e. produced PUs) were dissolved in DMSO-*d*_6_/*h*-DMSO at the concentrations of about 0.2 g/dm^3^. TMS was used as a standard. The proton spectra were employed additionally for the comparative polarity analysis of the polyurethanes which had no fluorine atoms in polymer chains. The study was based on the parameter *κ*_exp_ which was defined especially for that purpose. That parameter was calculated from the values of integrated signals in ^1^H NMR spectra of polyurethanes. The following protons were distinguished: those representing polar (*I*_***P***_) and apolar (*I*_***N***_) structural fragments within polyurethane chains with no fluorine atoms.

The factor *κ*_exp_ was calculated as:

6 $$ {\kappa_{\exp }}=\frac{I_P }{{I_P +{I_N}}}\cdot 100 \ \% $$

where:

7 $$ {I_P}=\sum {{I_{P_n }}={I_c}+0.5\left( {I_b +{I_{{b\prime }}}} \right)+{I_m}+{I_x}} $$

8 $$ {I_N}=\sum {{I_{N_n }}=} {I_i}+{I_j}+{I_h}+{I_{{h\prime }}}+{I_f}+{I_{{f\prime }}}+0.5\left( {I_b +{I_{{b\prime }}}} \right)+{I_d}+{I_k}+{I_l} $$

**Fig. 1:**
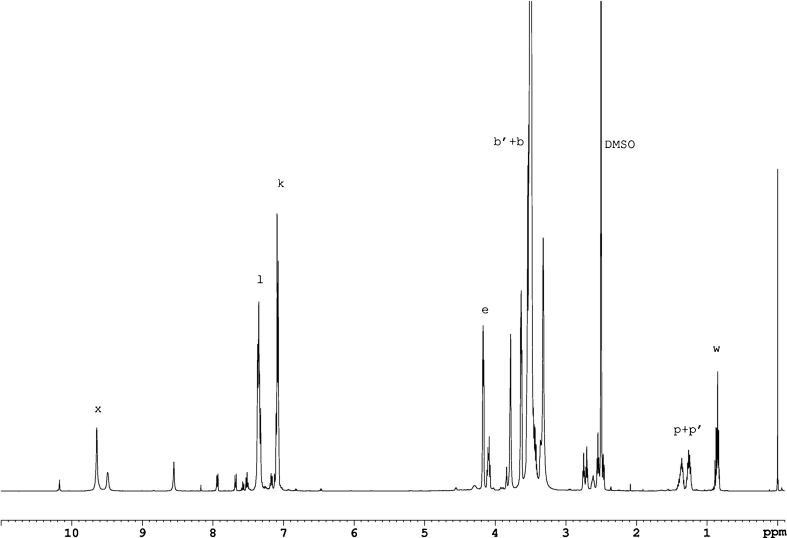
^1^H NMR spectrum for polyurethane No. 2, synthesised with the use of MDI diisocyanate

**Fig. 2:**
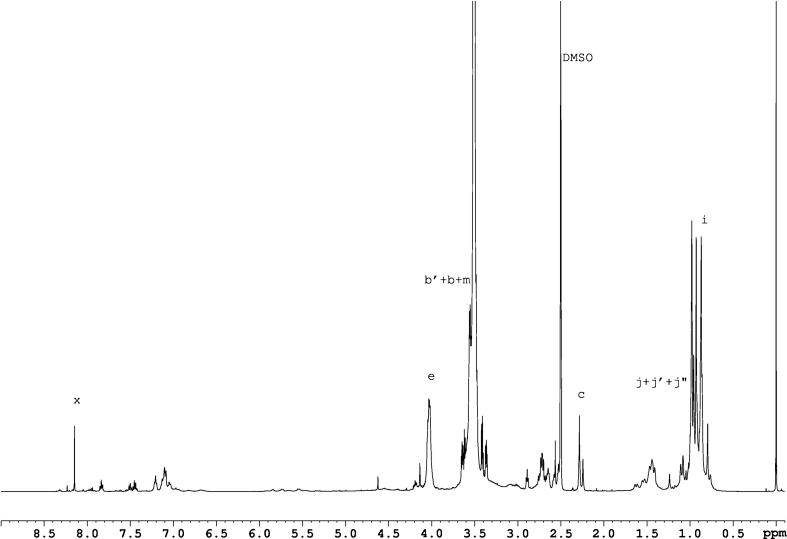
^1^H NMR spectrum for polyurethane No. 33, synthesised with the use of IPDI diisocyanate

For simplification, CH_2_–O (*b*) and CH_2_–N (*b*′) groups were assumed to have equivalent contributions to polar and nonpolar interactions. Table 2 provides the calculation method for the *κ*_exp._ values for exemplary samples (No. 2 and 33) from NMR spectra of those samples as presented in Figs. 1 and 2. The signals as recorded in those spectra were referred to protons designated with letters in Scheme 1.

**Table 2:** Analysis of signal integration in ^1^H NMR spectrum of the synthesised polyurethane No. 2 and 33

Type of structure	Based on NMR spectrum					Based on chemical formula (3)			
	Type of proton (Figs. 1 and 2 )	Sample No. 2		Sample No. 33		Type of proton	Structural fragment derived	Number of protons	
		*δ* (ppm)	Integrations (conventional unit)	*δ* (ppm)	Integrations (conventional unit)			Sample No. 2	Sample No. 33
Apolar structures	*I*_i_	–	–	0.77–0.88	1.6838	CH_3_–C	IPDI	–	18
	*I*_j_ + *I*_j′_ + *I*_j′_	–	–	0.93–1.24	2.8440	C–CH_2_–C	IPDI	–	12
	*I*_w_	0.83–0.91	0.3591	–	–	CH_3_–(CH_2_)_2_–CH_2_–N–	*N*-BDA	3	–
	*I*_p_ + *I*_p’_	1.23–1.40	0.5098	–	–	CH_3_–(CH_2-_ )_2_–CH_2_–N–	*N*-BDA	4	–
	*I*_c_	1.41–1.64	–	–	0.7890	CH_3_–(CH_2-_)_2_–CH_2_–N–	*N*-BDA	–	2
		–	–	2.24–2.28	0.3053	CH_3_–N	*N*-MDA	–	3
	0.5·(*I*_b_ + *I*_b’_ + *I*_m_)	3.32–3.4	12.5194	3.36–3.67	14.8291	O–CH_2_–CH_2_ –O (0.5 total amounts)	*N*-BDA	–	26
						O–CH_2_–CH_2_ –O (0.5 total amounts)	POG 2000	88	–
	*I*_d_	3.62–3.64	0.7310	–	–	Ar–CH_2_ –Ar	MDI	4	–
	*I*_k_ + *I*_l_	7.08–7.69	2.222	–	–	Ar	MDI	16	–
	*I*_N_		16.3413		20.4512			115	56
Polar structures	0.5·(*I*_b_ + *I*_b’_ + *I*_m_)	3.32–3.4	12.5194	3.36–3.67	14.8291	–CH_2_–NH–CO–	IPDI	–	4
						>CH–NH–CO–	IPDI	–	2
						O–CH_2_–CH_2_ –O (0.5 total amounts)	POG 600	–	26
						O–CH_2_–CH_2_ –O (0.5 total amounts)	POG 2000	88	–
						CH_3_–N–CH_2_ –	*N*-MDA	–	2
						C_3_H_7_–CH_2_–N	*N*-BDA	2	–
						CH_3_–N–CH_2_–	*N*-MDA	–	3
	*I*_e_	3.93–4.02	0.8607	4.01–4.05	1.0000	–CH_2_–O–CO–NH–	CH_2_ with urethan groups	8	8
	*I*_x_	9–10.5	0.4597	7.5–8.5	0.1479	–NH–CO–O–	Urethan	4	4
	*I*_P_		13.8398		15.9773			102	49
*κ*_theor_ (%)								47.0	46.7
*κ*_exp_ (%)		45.86		43.86					

*MDI* diphenylmethane diisocyanate, *IPDI* isophorone diisocyanate, *POG* polyoxyethylated polyols, *N-MDA N*-methyldiethanolamine, *N-BDA N*-butyldiethanolamine, *N-PhDA N*-phenyl-diethanolamine

In order to find the experimental *κ*_exp_ parameter, the integration values were analysed for all proton signals which were recorded within the NMR spectrum of a given sample. In particular, integrations of signals were distinguished for the protons which were present in functional groups and/or structural fragments with the polar and apolar characteristics. Independently, assuming the chain structure as per Eq. 3, the additional parameter of *κ*_theor_ was calculated as:

9 $$ {\kappa_{\mathrm{theor}}}=\frac{{\sum {n_i^{\mathrm{polar}}} }}{{\sum {\left( {n_i^{\mathrm{polar}}+n_i^{\mathrm{apolar}}} \right)} }} $$

where:

$$ n_i^{\mathrm{polar}} $$
Amount of protons in analysed polyurethane chain structures which were formally assumed as polar
$$ n_i^{{\mathrm{apola}{\mathrm{r}}}} $$
Amount of protons in structures which were formally assumed as apolar

The calculation method for the values of *κ*_theor_ was presented in Table 1; samples No. 2 and 33 were used as examples. Such analyses were not conducted for polyurethanes No. 9, 14 and 18, the synthesis of which involved TFBD since—as results from our earlier research [14]—the presence of fluorine atom(s) in the polyurethane chain makes the most decisive factor for the polarity of a polymer film. The *κ*_exp_ and *κ*_theor_ values for other polyurethanes can be found in Table 1. In the case of TFBD-extended polyurethanes No. 9, 14 and 18, such an analysis could not be carried out. The structures of those polymers were confirmed by FTIR, noting the presence of additional bands at 1,237.1, 1,201.7 and 1,116.2 cm^–1^, which were assigned to vibrations of C–F bonds. In Ref. [15], the authors demonstrated that the presence of fluorine atoms in the TFBD-extended PU chains could also be observed in ^19^F NMR spectra in which three groups of signals could be identified: *δ* = −120.93/−120.99, 123.33/−121.57 and −122.74/−122.93 ppm.

### Contact angle

Contact angles *Θ* were measured with the use of the method suggested by Zisman [16], i.e. by means of an optical goniometer with a digital camera installed in the axial direction of its lens. The liquid drops with the constant volumes (about 3–5 μdm^3^) were applied to the surfaces of the studied samples with the use of a special micropipette. The samples were fixed on the stage of the goniometer. The measurements were taken at 21 ± 1 °C. The contact angle values were found from the geometric analysis of pictures taken for liquid drops which involved the use of our originally developed software *Kropla* for interpretation.

### Surface free energy

Physical parameters of the surface energy of a solid *γ*_*S*_ were found on the basis of the Owens–Wendt method which assumes that the SFE *γ*_*S*,*L*_ may be presented as a sum of two components [13]:

10 $$ {\gamma_{S,L }}=\gamma_{S,L}^d+\gamma_{S,L}^p $$

where:

$$ \gamma_{S,L}^d $$
surface energy connected with dispersion interactions
$$ \gamma_{S,L}^p $$
surface energy connected with polar interactions

Equation 11 is generally applicable both to a solid phase, and the subscript of *S* is used then, and to a wetting liquid (standard liquid or tested solid material), with the subscript of *L*. The Owens–Wendt method was also convenient since it made it possible to evaluate the share of polar interactions in the total value of SFE, and thus it was possible to refer the values obtained for *γ*_*S*_ to the “amounts” of polar structures in linear polyurethanes (*κ*_exp_) as estimated from NMR spectra (Eq. 6).

The SFE for solids (*S*) and for liquids (*L*) interacting with those solids should satisfy the Owens–Wendt equation (Table 3):

**Table 3:** Surface properties of model measuring liquids [14]

Model measuring liquid	Surface free energy parameters (mJ/m^2^)		
	*γ*_L_	$\gamma_L^d$	$\gamma_L^p$
Water	72.8	21.8	51
Formamide	58.0	39	19
Diiodomethane	50.8	48.5	2.3

11 $$ {\gamma_L}\cdot \frac{{1+\cos \varTheta }}{2}=\sqrt{{\gamma_S^d\cdot \gamma_L^d}}+\sqrt{{\gamma_S^p\cdot \gamma_L^p}} $$

where *Θ* is the experimentally found contact angle between a liquid drop and a solid surface under investigation. So, wetting angles *Θ* were first measured for the surfaces of PU coatings with the use of two pair model liquids (water–diiodomethane and formamide–diiodomethane) with known parameters $\gamma_L, \ \gamma_L^d \ \mathrm{and} \ \gamma_L^p$. The obtained *γ*_*S*_ values were almost identical. The values shown in Table 5 refer to the formamide-diiodomethane system. Then, Eq. 11 was used to calculate the values $\gamma_S^p \ \mathrm{and} \ \gamma_S^d$ for the studied polyurethane films. The values *γ*_*S*_ were calculated from Eq. 10.

## Results and discussion

### Chemical structures of synthesised polyurethanes

Chemical structures of synthesised polyurethanes were presented in Scheme 1. NMR spectroscopy confirmed the presence of signals for expected protons. Figures 1 and 2 present the exemplary ^1^H NMR spectra for samples No. 2 and 33. Interpretations for those spectra were presented in Table 2.

**Fig. 3:**
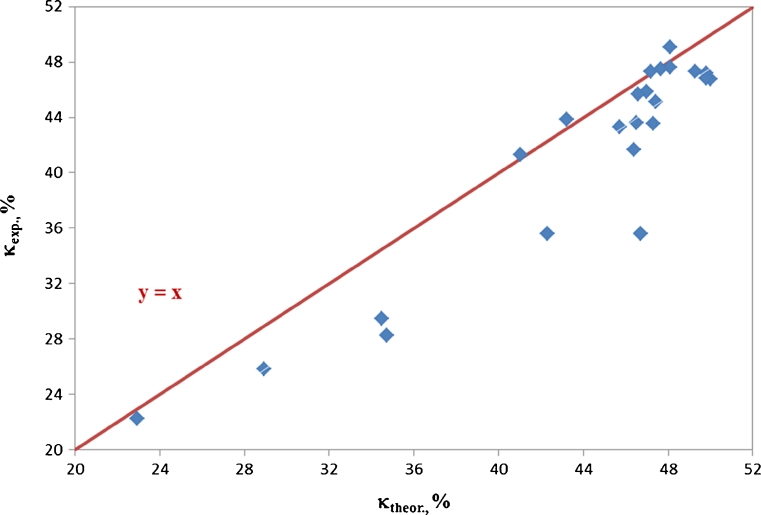
Illustration of the relation between *κ*_theor_ and *κ*_exp_ parameters for synthesised linear polyurethanes

The calculation procedure as adopted for the parameter *κ*_theor_ made it possible to give consideration to differences in molecular weights of polyols which were used in the synthesis: POG, PCL and PEA. For that purpose, the structures of those polyols were attributed the average numbers of structural repeating units (mers) which resulted from their molecular weight values. Thus, the parameter *κ*_theor_ could be used to evaluate the effect of polar interactions in the synthesised polyurethanes solely from the structural viewpoint. When chemical structures of obtained polyurethanes were perfectly in line with formula 5, the points with co-ordinates *κ*_theor_ and *κ*_exp_ should be situated on the straight line *y* = *x* as shown in Fig. 3 which presents the points with actual co-ordinates. The obtained regression equation $y=0.958x+0.282$ demonstrates that all specified points for which co-ordinate values were established within the real values of *κ*_theor_ are located below the straight line *y* = *x*, hence fewer polar structures are present in the synthesised polyurethanes than one could expect. That proves the systematic discrepancy between the chemical compositions of PUs as expected from stoichiometry and the compositions which are actually available from the synthesis. It is important since polar structures are formed generally as late as in the polyaddition process (e.g. bands of protons designated as *e* or *x*) while formally apolar structures appear even in isocyanate or hydroxyl parent substances. The parameters *κ*_exp_ and *κ*_theor_ were useful in the further part of the study to analyse the effect of the PU structure itself on SFE parameters of the polymer film obtained from that PU.

### SFE of the films which were obtained from synthesised polyurethanes

As results from our earlier research, SFE of polyurethane coatings is dependent on the polyurethane chemical structure which is strongly affected by a few factors, like: types of raw materials, mainly diisocyanates and polyols, conditions of the polyaddition process and conditions of the coating formation/application process. The coat formation (application) method is generally known to affect the orientation in domains which are composed of soft polyol segments and hard urethane segments; that orientation is decisive for the content of crystalline phase in those domains. Hence, the SFE value of polyurethane coats and films is influenced not only by the chemical structures of polyurethane chains but to some extent also by the polyurethane supermolecular structures. Those impacts result from the presence of hydrogen bonds, from polarity of segments and their susceptibility to dispersion interactions. Our earlier WAXS investigations revealed that the obtained (applied) polyurethane coats contained max up to 20 % of crystalline phase which was composed principally of hard segments [14]. It is hence the polyurethane chemical structure to be critical for SFE. An attempt was made initially to establish a correlation between SFE of the synthesised coats with their parameters *κ*_exp_ which—as comes from the analysis of results which was presented in Fig. 3—should make more reliable polarity indices than the parameters *κ*_theor_ (they were calculated from stoichiometry). Within over a dozen functions tested, the best match was observed for the linear dependence as below:

12 $$ {\gamma_S}=0.8737{\kappa_{\exp }}+0.5481 $$

**Fig. 4:**
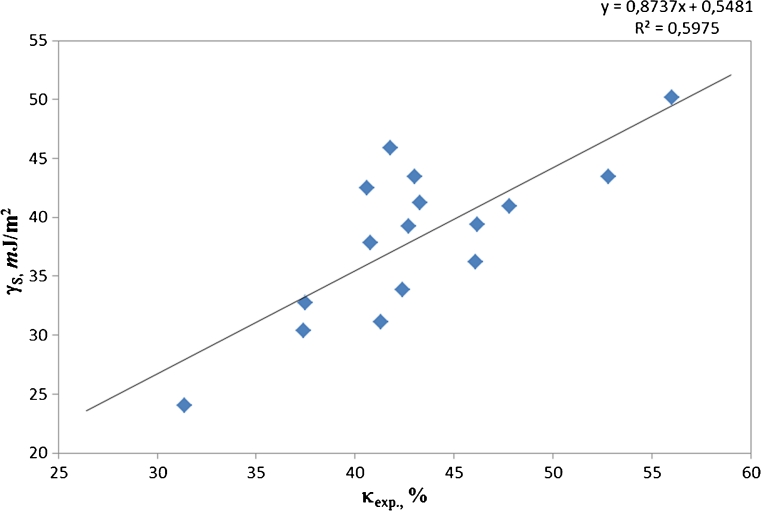
Correlations between the experimental SFE values (*γ*_*S*_) and *κ*_exp_ parameters calculated from ^1^H NMR spectra of synthesised polyurethane films without fluorine in polymer chains

The profile for that relation against the scattered measuring points was shown in Fig. 4. The linear trend (*R* ^2^ = 0.5975) follows the increasing values of those parameters pretty well but any prediction of the values for *γ*_*S*_ from the values of *κ*_exp_ may be pretty erroneous, in particular within the range *κ*_exp_ = 42–45 % which is most typical for the analysed polyurethanes. The SFE values within 30–45 mJ/m^2^ were measured for the films of those polyurethanes. That dispersion is too strong for the SFE values predicted solely on the basis of the parameter *κ*_exp_ to be correct. Whilst in the opinion of van Krevelen, other linear models with similar credibility may also be of value. An example of such a model is described on page 327 in Ref. [12]. That example is in line with the relation as suggested by Cotts and Reyes (1986): the dissipation factor tan *δ* versus dielectric constant *log*(ε)

13 $$ {\tan \delta } =\log \left[ {{{{\left( {0.1\cdot \varepsilon } \right)}}^5}} \right] $$

(*tan* δ and *ε* are to be measured at the same frequency).

### The additive model for SFE versus polyurethane structure

Having a relatively low credibility of a linear model in mind, we initiated the research programme to develop an empirical general model which should be capable of describing much better the relations between the polyurethane structures and SFE values of polymer films and coats obtained from those polyurethanes.

The fact was taken into consideration for the model(s) that the polyurethane structural criteria could most conveniently be presented by means of reactants which had been used in the synthesis of those polymers: the raw materials employed in the polyaddition processes control the structures and chemical compositions of the polyurethane products. The effects which are also important come from the process conditions, from the method adopted in the polymerisation process, and—as demonstrated in the studies—from the physical structures of the obtained coat surfaces. However, not all parameters could, be taken into consideration since some of them could not be represented quantitatively. The fact that the substrate types were assumed as independent variables should be seen as a purely formal manipulation which made it possible to give consideration to polyurethane structures. Two model types were initially taken into consideration:

- A model in which additivity of the components in the function *γ* was assumed, hereinafter “additive model” (it was found more useful in the research presented in this paper), and
- A model with the multiplicative function *γ*—“multiplicative model” (that was employed, e.g. in modelling the substitution effect during polymerisation, when the total kinetic equation constant is determined as a product of components which are dependent on the mer conversion degrees [17]).

A sum of component arguments is thus used in the additive model to find a variable value while a product of those components is used in the multiplicative model. The possibility of linking the SFE to the parachor parameter, and the additive effects of the SFE components, made us select the additive model as a tool for the description of the effect(s) of the polyurethane chemical structure(s) on the SFE values of the produced polymer film(s).

The present study was based on the Owens–Wendt model (Eq. 10) which assumes that the SFE is an additive quantity as regards polar and dispersive interactions. The SFE was presented in the additive model developed herein as a sum of individual substrate-dependent components and a constant term (independent on said components).

The following steps were required to build our model:

1. Categories of variable input data were defined which would affect the value of *γ*_S_. Those categories were related to substrate types in this case. Each category was formed by a set of substrates which had been used in the synthesis, i.e. diisocyanates, polyols and low molecular weight compounds which were employed as prepolymer chain extenders.
2. Successive integers were then assigned at random to individual elements within each set. Sets which “remembered” or “stored” numbers for substrates instead of their names were created in this way. These were the sets, in succession: diisocyanates (*I*), polydiols (*J*) and chain extenders (*Q*).

Those data sets may be presented as follows:

14 $$ \begin{array}{*{20}{c}}
   {I = \left\{ {1,2,3, \ldots  \ldots x} \right\}} \hfill  \\
   {J = \left\{ {1,2,3, \ldots  \ldots y} \right\}} \hfill  \\
   {Q = \left\{ {1,2,3, \ldots  \ldots z} \right\}} \hfill  \\
\end{array} $$

where:

*x*, *y* and *z* stand for the numbers of elements in each substrate set, [elements] assigned to the respective category, while the *i*th element within the set *I*, *j*th element within the set *J* and *k*th element within the set *Q*, these represent the number of a specific substrate.

3. Assuming that each substrate brings its contribution to the value of *γ*_*S*_, one may create successive sets *A*, *B*, and *C*, which will “store” the contribution values for each raw material. In that case, the searched mathematical model will have the form of a complex function:

15 $$ {\gamma_S}=A\left[ i \right]+B\left[ j \right]+C\left[ k \right]+\mathrm{const} $$

where:

*A*, *B* and *C* are discrete functions which link each item within the substrate set *I*, *J* and *Q* with their corresponding values which represent the contributions of those substrates to the value *γ*_S_.

The numerical estimation of parameters in the model 15 consists in adjusting the values of all variables *A*[*i*], *B*[*j*] and *C*[*k*] in such a way as to minimise the absolute deviation values between the calculated and experimental values, which may also be seen as absolute error of calculated values. Thus, minimisation of the deviation values makes an objective function of that estimation. The minimum for deviations in estimated parameter values against experimental values for the whole data set may be defined in various ways, inter alia as the minimum of the arithmetical average of errors, minimum of median or minimum of the maximum deviation in the set of all deviations. If minimisation of the arithmetical mean of the deviation values is assumed as the objective function, one aims at the best match between the estimated and experimental values, but one should be prepared at the same time for essential differences between the minimum and maximum deviations. The distribution of error values, if analysed, will identify gross errors in that case, i.e. clearly isolated values. Such blunders may result from various reasons which may sometimes be hard to identify, like ordinary calculation errors, incorrectly conducted syntheses and/or SFE determinations, different chemical compositions of polyurethanes, essential differences in the physical structures of the applied coats, etc. When a few measurement results are available for the same set of initial data, that method makes it possible to reject unreliable findings. In case of a single measurement point only, a decision may be taken to repeat that measurement when needed, which we did a few times.

We found out within our further studies, with the use of the statistical analysis, that the distribution of deviations in the version with minimisation of the arithmetic mean of errors was not a normal distribution. Thus, the arithmetic mean was not a correct measure to characterise the mean value in that distribution. A median was hence decided to be used for that purpose. Minimisation of the median value became the objective function in that version. Alike for the case of minimisation of the arithmetic mean error, suggestions were also possible here on the results loaded with gross errors: repeated measurements for certain points and/or rejection of some incorrect results.

If the minimum value of the maximum deviation between the calculated and experimental data was assumed as the objective function, then its minimisation would give the estimated parameter values which would make it possible to reduce the span between the minimum and maximum results. Yet, that type of optimisation would yield the “side” effect—no information available on the extreme/isolated results. There would be no grounds hence to reject the strongly erroneous results, both resulting from measurement and calculation errors, since the error values would be averaged in that case.

The search for suitable parameters was initiated from an initial point which was selected at random. The random function values *A*, *B* and *C* were specific for that point. The values of those estimated parameters were then changed to minimise the objective function value. As it was impossible to find the global minimum for the objective function in that method, estimation procedures were repeated many times, starting from any initial point, i.e. from the randomised set of initial parameter values *A*[1, 2, …, *x*], *B*[1,2, …, *y*] and *C*[1,2, …, *z*]. The obtained sets of estimated parameters were stored in back-up files. Such calculations were repeated many many times, going to 1,000 repetitions. After the estimation cycle was completed, the archived deviation values for individual estimations were compared against each other to finally select the set of parameter which offered the best match with the experimental findings.

The individual estimation was conducted as follows: after defining the set of initial parameters, a set of values $\gamma_S^{\mathrm{estim}}$ was determined which corresponded to the set of experimental data $\gamma_S^{\exp }$. Each experimental value *γ*_*S*_ was compared with experimental result, and a set of deviations was created $\left| {\gamma_S^{\exp }-\gamma_S^{\mathrm{estim}}} \right|$, and subsequently the average value of absolute deviations was calculated (arithmetical average or median, respectively). Then, one of parameters: *A*[*i*], *B*[*j*] or *C*[*k*] was selected at random and its value was changed until the average value of deviations was reduced for the whole set. Sampling and estimation of individual parameters was repeated until the estimation procedure yielded no further change in any of those parameters. Finally, the value of the constant parameter (const) was subjected to estimation.

The calculation time within individual estimation was principally dependent on the number of experimental data points and on the number of parameters subjected to estimation, and it varied between a few and over a dozen minutes. A typical estimation which involved 1,000 repetitions usually took 10–15 days, with the use of a typical PC.

Table 4 presents the codes adopted for categories of the independent variables and the polyurethane synthesis substrates which belong to those categories. Substrates were initially assigned any codes of independent variables which had to be put in order at successive estimations to provide subsequent legibility of the presented model diagrams. The goal was to avoid overlapping of data points in the presented diagrams.

**Table 4:** Polyurethane raw materials denotation (after final arrangement)

Category of the independent variable	Type of substrate	The substrate attributed to independent variable	The value of the independent variable in this category
*I*	Diisocyanate	MDI	1
		TDI	2
		HDI	3
		IPDI	4
*J*	Polyol	POG 2000	1
		POG 600	2
		PEA 1000	3
		PCL 2000	4
		PCL 530	5
		PTMO 1000	6
*K*	Chain extender	TFBD	1
		*N*-BDA	2
		*N*-PhDA	3
		*N*-MDA	4
		HD	5
		BD	6
		*N*-*tert*-BDA	7

*MDI* diphenylmethane diisocyanate, *TDI* toluene diisocyanate, *HDI* hexamethylene diisocyanate, *IPDI* isophorone diisocyanate, *POG* polyoxyethylated polyols, *PEA* poly(ethylene adipate), *PCL* polycaprolactone, *N-MDA N*-methyldiethanolamine, *N-BDA N*-butyldiethanolamine, *N-PhDA N*-phenyl-diethanolamine, *BD* 1,4-butanediol, *HD* 1,6-hexanediol, *TFBD* 2,2,3,3-tetrafluoro-1,4-butanediol, *N*-*tert*-butyldiethanolamine (*N*-*tert*-BDA)

The early analyses demonstrated no essential relation between the SFE values and the type of solvent (1,4-dioxane or chlorobenzene) in which the isocyanate prepolymer chain extension reactions were conducted with the use of low molecular weight diols, and which were used to form the coats to be tested. Hence, no impacts from solvents were taken into consideration in further calculations.

**Fig. 5:**
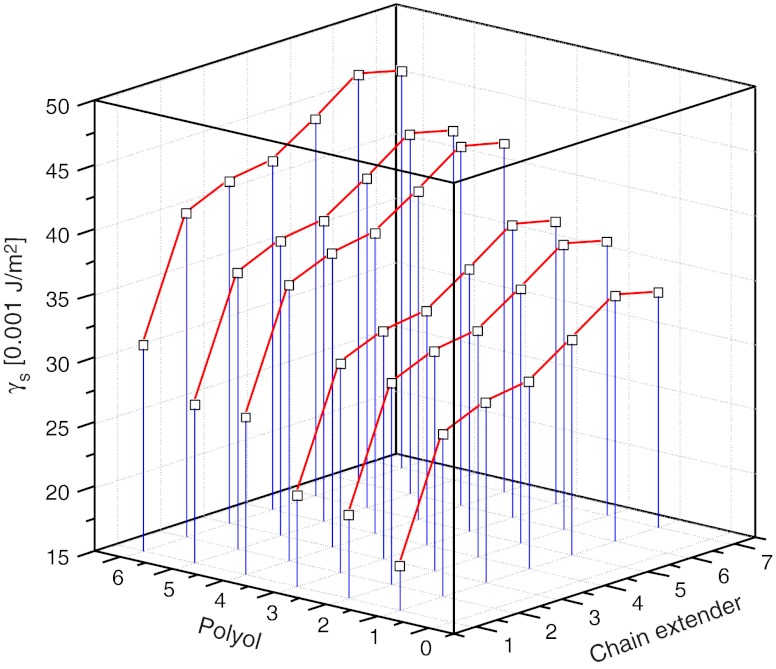
SFE profiles for PU coats synthesised from MDI (*i* = 1) versus type of polyol and chain extender

**Fig. 6:**
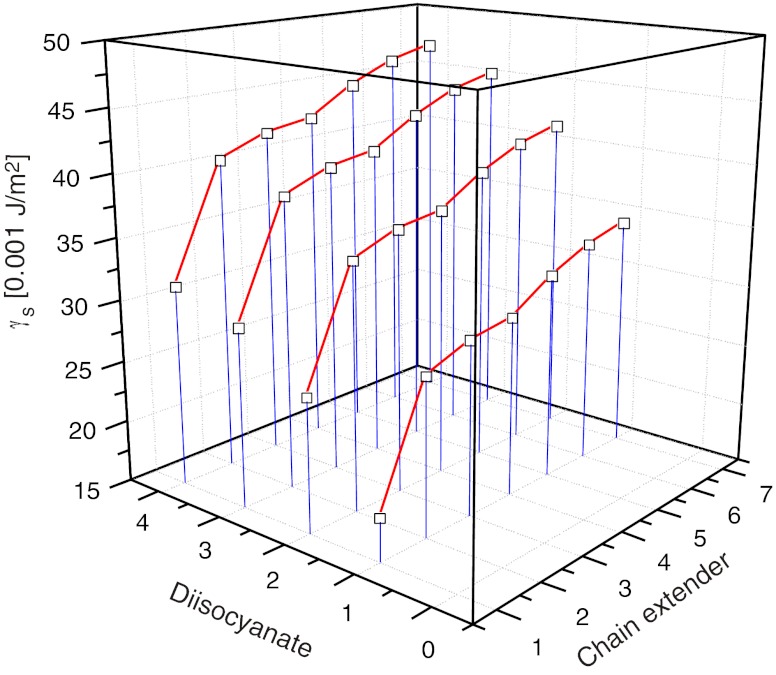
SFE profiles for PU coats synthesised from POG 2000 (*j* = 1) versus type of diisocyanate and chain extender

Table 5 shows the SFE values which were obtained from initial numerical calculations. No values of the estimated parameters: *A*, *B*, *C* and *const* were analysed at that stage. The obtained data, however, made it possible to reject highly erroneous findings, most probably erroneous by incident, for repeated combinations of the same substrates (here, the determination results were used as $\gamma_S^{\exp }$ for PU coats obtained from 1,4-dioxane or chlorobenzene solutions). However, repeated measurements were decided to be required for highly erroneous results (*italic type*). Repeated syntheses and repeated measurement of wetting angle values *Θ* were conducted for those combinations of substrates (i.e. for those samples). Based on them, new values of $\gamma_S^{\exp }$ were calculated—as presented in Table 6. The results were closer to those initially estimated in three cases. The results of subsequent estimation, with due consideration of the data in Table 6, were presented in Table 5. The estimated parameter values for the model were specified in Table 7. They made a basis for the 3D diagrams which illustrated the changes in SFE of polyurethane coats versus their chemical compositions. Examples of diagrams were provided in Figs. 5 and 6.

**Table 5:** SFE values for polyurethane films obtained from numerical calculations

Sample No.	The value of the independent variable for *i* category	The value of the independent variable for *j* category	The value of the independent variable for *k* category	Estimation No. 1			Estimation No. 2		
				$\gamma_S^{\exp }$.(mJ/m^2^)	$\gamma_S^{\mathrm{estim}}$ (mJ/m^2^)	Relative error (%)	$\gamma_S^{\exp }$ (mJ/m^2^)	$\gamma_S^{\mathrm{estim}}$.(mJ/m^2^)	Relative error (%)
1	1	1	2	37.72	27.59	26.86	Decline		
2	1	1	2	27.59	27.59	0.00	27.59	27.59	0.00
3	1	1	3	29.00	29.00	0.00	29.00	29.00	0.00
4	1	1	4	30.39	30.39	0.00	30.39	29,51	2.90
5	1	1	4	29.51	30.39	2.98	29.51	29,51	0,00
6	1	1	6	33.19	33.19	0.00	33.19	33.24	0.15
7	1	1	6	33.19	33.19	0.00	33.19	33.24	0.15
8	1	1	7	34.07	34.07	0.00	34.07	34.07	0.00
9	1	2	1	21.47	21.47	0.00	21.47	21.47	0.00
10	1	2	4	31.53	31.53	0.00	31.53	32.55	3.24
11	1	2	5	42.37	35.05	17.28	36.78	34.73	5.57
12	1	3	4	37.74	37.74	0.00	37.74	33.16	12.14
13	1	4	6	42.03	42.03	0.00	42.03	42.03	0.00
14	1	5	1	27.28	27.28	0.00	27.28	27.28	0.00
15	1	5	6	42.03	40.14	4.63	42.03	42.03	0.00
16	1	6	6	45.80	45.80	0.00	45.80	45.80	0.00
17	2	1	6	40.54	40.54	0.00	40.54	40.50	0.10
18	2	2	1	34.87	28.82	17.35	28.73	28.73	0.00
19	2	2	6	40.45	41.68	3.04	40.54	43.54	7.64
20	3	1	2	38.60	38.60	0.00	38.60	38.60	0.00
21	3	1	2	45.81	38.60	15.74	Decline		
22	3	1	2	37.44	38.60	3.10	37.44	38.80	3.10
23	3	1	6	44.30	44.20	0.23	44.30	44.25	0.11
24	4	1	2	29.33	38.20	30.24	Decline		
25	4	1	2	43.46	38.20	12.10	43.46	40.42	6.99
26	4	1	4	41.00	41.00	0.00	41.00	42.34	3.27
27	4	1	5	38.89	44.52	14.48	Decline		
28	4	1	5	47.65	44.52	6.57	47.65	44.52	6.57
29	4	1	5	44.52	44.52	0.00	44.52	44.52	0.00
30	4	1	6	50.15	43.80	12.66	50.15	46.07	8.14
31	4	1	6	46.07	43.80	4.93	46.07	46.07	0.00
32	4	2	4	38.11	42.14	10.57	45.32	45.38	0.13
33	4	2	4	47.45	42.14	11.19	47.45	45.38	4.36
34	4	3	4	41.03	48.35	17.84	41.03	45.99	12.09

**Table 6:** SFE values for repeated synthesis PU films

Sample No.	Substrates			$\gamma_S^{\exp }$ (mJ/m^2^)
	Type of diisocyanate	Type of polyol (molecular weight)	Type of chain extender	
11	1	2	5	36.78
18	2	2	1	28.73
32	4	2	4	45.32
34	4	3	4	36.91^a^

^a^The result with more bigger error remains decline

**Table 7:** Parameters for additive model for SFE versus polyurethane structure calculated in numerical estimation No. 2

The value of the independent variable	*A*	*B*	*C*
1	9.58	0.13	8.72
2	16.84	3.17	17.88
3	20.59	3.78	19.29
4	22.41	8.92	19.8
5		8.98	21.98
6		12.69	23.53
7			24.36
Const = 0.00			

*Const* constant parameter

When analysing the obtained relations, one should remember that they relate to the PU chemical structures as defined by formula 5, and in particular to elastomer amorphous films or to those containing up to 20 % crystalline phase. The question is less important which solvent was employed to prepare the process solutions from which the polymer films were then formed.

### Analysis of effects of polyurethane structures on SFE values of obtained polymer films

The effects of polyurethane structures on the SFE values of applied polymer films were analysed on the basis of the data obtained from estimation No. 2 (Table 5) as well as the relations which resulted from model 15 and diagrams plotted for it. The raw materials shown in diagrams determine, as discussed above, the nature of chemical structures in polyurethane chains as shown in Scheme 1. Credibility of the obtained results was mostly dependent on the number of experimental data points and on reliability of the points which made the basis for estimation of the parameters in the additive model 15. A relatively low number of available experimental data, and measurement errors at the stage of polyurethane synthesis—as shown inter alia by the differences in *κ*_theor_ and *κ*_exp_ values presented in Fig. 3—and errors in determinations of SFE values, restrict credibility of the model 15 considerably. Nevertheless, attempts can be made on the basis of that model to develop generalisations which would be useful in predicting the effect of polyurethane composition on the polyurethane surface properties which are important in developing protective coats.

- When the raw materials needed for the production of linear polyurethanes are selected adequately, it is possible to obtain extensive structural changes which induce changes in SFE of polymer films within 18 to 50 mJ/m^2^. That is a very wide span of SFE which suggests that this method may be applicable in the production of various materials, from apolar films or coats to materials with considerable polarity.
- The models show that the change in the diisocyanate component from *i* = 1 to 4 (Table 4) increases the SFE value by *Δ* = 10–15 mJ/m^2^ if the chain extender (Fig. 6) or polyol component remain the same. Samples No. 6, 17, 23 and 30 are examples of such polyurethanes; those were synthesised with the use of such diisocyanate feeds as POG 2000 polyol (*j* = 1) and BD chain extender (*k* = 6) which are in common practice. That makes an interesting observation which is in line with the calculated values of the *κ*_theor_ parameter for the coats obtained from MDI, TDI and HDI (samples No. 6, 17, 23). The result for sample No. 30 (*κ*_theor_ = 46.5 %) would suggest, on the other hand, that the IPDI-based coats should be more hydrophobic than found in SFE measurements.
- The diagrams in Fig. 5 indicate that a change in the polyol component from *j* = 1 to *j* = 7 may increase the SFE values. Films produced with the use of polyesters PCL 530 or PEA 1000 (samples No. 15 and 12) are more polar and hydrophilic than their POG-2000 based counterparts (samples No. 6 and 5). The observed trend is completely in line with the values *κ*_exp_ as found by the ^1^H NMR spectroscopic method. The *κ*_exp_ parameters for poly(ester-urethanes) (samples No. 12 and 15) are much higher than those for more hydrophobic samples No. 5 and 6 (Table 1). However, no confirmation was obtained for the opinion, which was derived principally from the parameter *κ*_theor_, that the increasing molecular weight of polyol POG from 600 to 2,000 g/mol and PCL from 530 to 2,000 g/mol should cause a considerable increase in the SFE value. For example, the molecular weight values for polyester polyol (PCL; *j* = 4, 5) and for polyether polyol POG had no essential impacts on the SFE values in case of MDI-derived polyurethanes.
- The most important effect on the chemical nature of polyurethane coats comes from the type of the chain extender. TFBD as a fluorine-containing reactant offered the most prominent effect within the tested substrates. That substrate contributes to the formation of coats with the definitely highest hydrophobicity (Fig. 5). Figure 5 indicates, that the use of MDI and POG 2000 is most favourable for the production of fluorine-containing hydrophobic coats. That is completely in line with our earlier observations [18]. When BD is substituted by HD or *N*-MDA, the SFE value will be lower by about *Δ* = 10-12 mJ/m^2^, and further shift to *N*-BDA (*k* = 2) will result in further decline of SFE by a similar value. On the other hand, relatively high SFE values for polyurethanes synthesised with *N*-*tert*-BDA result from strong dispersion interactions which are specific for a tertiary alkyl substituent. Those interactions increase the component $\gamma_S^d$ (in Eq. 10) which has been confirmed in [19].

## Conclusions

As results from the conducted studies, polyurethane coats offer a wide span of changes within SFE determined by the Owens–Wendt method on the basis of measured contact angle values. There exists a possibility to forecast the approximate SFE values for polyurethane films; the forecasts are based on the parameter *κ*_exp_ which is to be found by comparing integrations for selected signals in ^1^H NMR spectra. That parameter correlates pretty well with the parameter *κ*_theor_ which can be calculated from the PU chain structure on the condition that the synthesised coats are generally amorphous or that they contain below 20 % of crystalline phase.

The SFE values can also be predicted on the basis of the adequately compiled additive models which are analogous to those developed by van Krevelen and which indirectly consider the effect of polyurethane structures through the basic substrates used in the synthesis: diisocyanates, polyols and chain extenders. The model calculations were based on minimisation of the average deviations in SFE values which had been estimated numerically in models against the experimental values. That approach made it possible to verify the initial experimental values which had gross errors. The additive models were then developed and they confirmed that the SFE values for polyurethanes may be modified over a wide span: 20–50 mJ/m^2^ while the specific value may be controlled by the PU structure which is defined by the type of initial diisocyanate, polyol, and isocyanate prepolymer chain extender. The use of TFBD in that capacity was found to yield apolar coats in practice which results from the presence of fluorine atoms incorporated into the polyurethane chains at the level of about 6 wt%. The effect of polyol is not so important in that case. As expected, polyesters promote formation of hydrophilic coats more than polyethers do, but the molecular weight value of a polyol is definitely of lower importance. The models developed need to be further expanded by adding the findings for polyurethane ionomers; these are planned to be presented in part 2 of this paper, to make the models applicable in designing materials for bio-outlets and for protective coatings.
